# The complexity of uncertainty: lessons from chimerism in in vitro fertilization

**DOI:** 10.1016/j.xfre.2026.02.004

**Published:** 2026-02-19

**Authors:** Greysha Rivera-Cruz, Eduardo Hariton

**Affiliations:** a Division of Reproductive Endocrinology and Infertility, Department of Obstetrics and Gynecology, Stanford University, Stanford, California; b Reproductive Science Center of the San Francisco Bay Area, Oakland, California

Rare genetic phenomena challenge not only our understanding of human development but also the frameworks through which reproductive medicine interprets risk, responsibility, and outcome. Because fertility care increasingly incorporates expanded genetic testing into routine evaluation, clinicians are encountering results that are biologically possible yet clinically indeterminate. These incidental findings often resist straightforward interpretation and complicate reproductive counseling in ways that extend beyond the original intent of testing. The case reported by Patel et al. (1), describing a suspected case of congenital chimera conceived through assisted reproductive technologies (ARTs), offers a compelling illustration of this emerging reality.

Patel et al. (1) reported an interesting case of an incidental possible chimera after identifying an extra Y-chromosome material on a healthy-appearing individual following an inconclusive carrier screening result. The patient was conceived through ARTs after the transfer of three embryos, leading to a dizygotic twin gestation that resulted in the delivery of the patient and her phenotypically healthy brother. Of note, it is important to establish that a chimera diagnosis should be made through cytogenetic analysis or long-read genomic sequencing, not with next-generation sequencing data. While this case is interesting, we cannot definitively say that this is a human chimera case, as not all 46,XX/46,XY cases represent a chimera. There are plausible mechanisms by which these finding can also be seen; unique chromosomal rearrangements/translocations as well as mosaic karyotype. While the biologic mechanisms underlying chimerism are increasingly well described, its suspicion in this context underscores a broader issue in contemporary fertility practice: genetic testing is now capable of revealing complexity that often exceeds our ability to assign clinical meaning, including reproductive outcomes.

Chimerism and mosaicism are distinct phenomena that are frequently confused, particularly in the context of ART. Mosaicism refers to the presence of two and occasionally more, genetically different cell lineages originating from the same zygote usually as a result of mitotic errors; whereas chimerism denotes the coexistence of two or more cell lineages that originate from distinct zygotes (2). Although the definitions may appear similar, their underlying causes are fundamentally different. Chimerism can also be acquired as a result of twin–twin cell transfer in utero, bone marrow transplantation, or maternal–fetal cell exchange during pregnancy, also known as microchimerism (2, 3). Patel et al. (1) clearly outline these mechanisms and situate their case within the historical context of multiple embryo transfer practices that were once common in in vitro fertilization (IVF).

Rather than reteaching mechanisms, the most salient contribution of this case lies in what it reveals about the evolving landscape of incidental findings in reproductive genetics. Chimerism in this setting was not sought, suspected, or clinically indicated before testing; it was suspected after the test, which is designed for carrier risk assessment, detected an unexpected signal (1). Expanded carrier screening was developed to identify individuals or couples at increased risk for recessive and X-linked disorders, not to diagnose complex genomic events, resolve questions of embryologic origin, or predict long-term reproductive outcomes. As we have broadened the number of genes included in expanded carrier screening, we have observed an expected increase in the incidence of positive results for manifesting carriers. This illustrates that while the initial purpose of this testing was to serve as a screening tool for recessive disorders, it now also identifies individuals with pathogenic variants in genes that exhibit both dominant and recessive inheritance patterns. Examples include cancer predisposition genes such as Ataxia Telangiectasia Mutated (*ATM*), Bloom Syndrome RecQ Like Helicase (*BLM*), and Fumarate Hydratase (*FH*), among others. However, as testing continues to expand, these secondary findings are becoming increasingly unavoidable.

In vitro fertilization settings are particularly susceptible to this challenge. Fertility clinics routinely employ genetic testing across multiple stages of care, including carrier screening, preimplantation genetic testing for monogenic disorders (PGT-M), and prenatal screening. The density of testing, combined with heightened scrutiny of outcomes and expectations of precision, amplifies the likelihood that incidental findings will be identified. Patel et al. (1) describe practical downstream implications if a confirmed case of human chimerism, including challenges with linkage analysis for PGT-M, limitations of prenatal screening and diagnostic testing because of mixed cell populations, and broader issues related to kinship analysis and germline interpretation. Chimerism therefore represents an archetype of the modern incidental finding: biologically possible, diagnostically challenging, and often only partially actionable.

The phenotypic spectrum of human chimeras is diverse, ranging from fetuses and newborns with congenital anomalies and disorders of sexual development to milder presentations, including males with oligospermia and otherwise healthy individuals, as illustrated by the patient in this case (4, 5). Importantly, even advanced genomic tools may fail to capture the full biologic picture, as the proportion and distribution of genetically distinct cell lines can vary substantially across tissues (1, 2). Consequently, results may be discordant, incomplete, or misleading when interpreted outside the intended context of the test. This creates an expected clinical tension: the understandable desire for diagnostic closure may prompt additional testing, expert consultations, and prolonged uncertainty without necessarily improving reproductive decision-making.

This tension is not unique to chimerism. A similar situation is seen in prenatal genetics, where cell-free fetal deoxyribonucleic acid screening tests return inconclusive results after detecting multiple chromosomal abnormalities, raising the risk of maternal malignancy in approximately 40% of cases (5). The complexity of interpreting these results, coupled with the broad spectrum between pathologic findings and normal biologic variation, raises complex questions about counseling, downstream evaluation, and the balance between potential benefit and harm (5). In both scenarios, advances in testing reveal biologic complexity that outpaces our interpretive frameworks.

What, then, can we learn from chimerism in IVF?

First, deeper genetic resolution does not necessarily confer greater clinical clarity. Precision medicine, particularly in reproductive care, inevitably exposes biologic variation that defies binary classification. Second, incidental findings are not failures of testing; they are a predictable consequence of expanded approach to testing. The challenge lies less in preventing discovery and more in preparing clinicians and patients to navigate implications responsibly.

Pretest counseling is therefore central. Counseling around expanded carrier screening and other genetic tests should explicitly address the possibility of incidental findings, including results of uncertain significance or discoveries unrelated to the original testing indication, as well as actionable manifesting carrier results. Patients should be counseled that some findings may necessitate further evaluation without yielding definitive answers, and that uncertainty may persist despite exhaustive investigation (2). This is not pessimism; it is informed consent aligned with the realities of modern genomics.

Posttest management should likewise prioritize proportionality. Not every unexpected result warrants escalation, invasive testing, or reinterpretation of reproductive outcomes. Early involvement of reproductive genetic specialists can help contextualize findings, set realistic expectations, and reduce avoidable anxiety. Patel et al. (1) model this approach by outlining counseling on the limitations of proceeding without interpretable carrier screening, the implications for PGT-M, and the need to anticipate testing challenges during pregnancy.

Finally, this case invites reflection on how IVF outcomes are defined and perceived. As genetic testing becomes more comprehensive, there is a risk of over-medicalizing variation that falls within the spectrum of human biology. Chimerism challenges simplistic narratives of success and failure in ART and reminds us that biologic complexity does not equate to pathology. It also supports a broader point: uncertainty is not an aberration in reproductive genetics; it is an inherent feature of practice in this genomic era.

In an age where genomic information is increasingly abundant, clinicians and patients need shared language for uncertainty systems that support thoughtful interpretation rather than reflexive diagnostic escalation. The case presented by Patel et al. (1) provides a timely and instructive lens through which to reconsider how reproductive medicine engages with incidental genetic findings.

## CRediT Authorship Contribution Statement

**Greysha Rivera-Cruz:** Writing – original draft, Writing – review & editing. **Eduardo Hariton:** Conceptualization, Writing – original draft, Writing – review & editing.

## Declaration of Interests

G.R. has nothing to disclose. E.H. has nothing to disclose.
